# Characterization of anti-erythrocyte and anti-platelet antibodies in hemolytic anemia and thrombocytopenia induced by *Plasmodium* spp. and *Babesia*spp. infection in mice

**DOI:** 10.3389/fcimb.2023.1143138

**Published:** 2023-04-14

**Authors:** Mo Zhou, Jun Xie, Osamu Kawase, Yoshifumi Nishikawa, Shengwei Ji, Shanyuan Zhu, Shinuo Cao, Xuenan Xuan

**Affiliations:** ^1^ National Research Center for Protozoan Diseases, Obihiro University of Agriculture and Veterinary Medicine, Obihiro, Hokkaido, Japan; ^2^ Jiangsu Key Laboratory for High-tech Research and Development of Veterinary Biopharmaceuticals, Jiangsu Agri-animal Husbandry Vocational College, Taizhou, China; ^3^ Engineering Technology Research Center for Modern Animal Science and Novel Veterinary Pharmaceutic Development, Jiangsu Agri-animal Husbandry Vocational College, Taizhou, China; ^4^ Department of Biology, Premedical Sciences, Dokkyo Medical University, Tochigi, Japan

**Keywords:** malaria, Babesiosis, thrombocytopenia, anemia, anti-erythrocyte auto-antibodies, anti-platelet auto-antibodies

## Abstract

**Introduction:**

Malaria and Babesiosis are acute zoonotic disease that caused by infection with the parasite in the phylum Apicomplexa. Severe anemia and thrombocytopenia are the most common hematological complication of malaria and babesiosis. However, the mechanisms involved have not been elucidated, and only a few researches focus on the possible role of anti-erythrocyte and anti-platelet antibodies.

**Methods:**

In this study, the *Plasmodium yoelii, P. chabaudi, Babesia microti* and *B. rodhaini* infected SCID and ICR mice. The parasitemia, survival rate, platelet count, anti-platelet antibodies, and the level of IFN-γ and interleukin (IL) -10 was tested after infection. Furthermore, the *P. yoelii, P. chabaudi, B. rodhaini* and *B. microti* infected ICR mice were treated with artesunate and diminaze, the development of the anti-erythrocyte and anti-platelet antibodies in chronic stage were examined. At last, the murine red blood cell and platelet membrane proteins probed with auto-antibodies induced by *P. yoelii, P. chabaudi, B. rodhaini*, and *B. microti* infection were characterized by proteomic analysis.

**Results and discussion:**

The high anti-platelet and anti-erythrocyte antibodies were detected in ICR mice after *P. yoelii, P. chabaudi, B. rodhaini, and B. microti infection*. Actin of murine erythrocyte and platelet is a common auto-antigen in *Plasmodium* and *Babesia* spp. infected mice. Our findings indicate that anti-erythrocyte and anti-platelet autoantibodies contribute to thrombocytopenia and anemia associated with *Plasmodium* spp. and *Babesia* spp. infection. This study will help to understand the mechanisms of malaria and babesiosis-related thrombocytopenia and hemolytic anemia.

## Introduction

The intraerythrocytic apicomplexan parasites *Plasmodium* and *Babesia* spp. cause malaria and babesiosis, respectively. Malaria and babesiosis are accountable for significant mortality and morbidity to humans and animals globally (Zoleko Manego et al., 2019; Zottig and Shanks, 2021; Zowonoo et al., 2023). Thrombocytopenia and anemia, are common symptoms of malaria and *Babesia* spp. infection (Yuan-Yuan et al., 2016; Yu et al., 2021; Waked and Krause, 2022). It has been reported that acute malaria infection is associated with autoimmune hemolytic anemia (AIHA), but it has not been well characterized. The symptom of AIHA include shortened red blood cells (RBCs) survival as well as the autoantibodies found in direct antiglobulin tests (DATs). DATs test complement C3d and/or immunoglobulins against autologous RBCs (Santos et al., 2020; Rajapakse and Bakirhan, 2021; Sporn et al., 2021; Ueda, 2022).

Anemia and thrombocytopenia are the most common hematological complications of malaria and babesiosis. Several studies have documented a high rate of thrombocytopenia in malaria patients. Over the past four decades, research has been conducted on the malaria thrombocytopenia, however, the exact mechanism behind this phenomenon remains unclear (Zumla and Hui, 2019; Tao et al., 2020; Tołkacz et al., 2021; Vanheer and Kafsack, 2021; Voisin et al., 2021; Zottig and Shanks, 2021). Thrombocytopenia in malaria is a multifactorial phenomenon likely caused by increased platelet destruction and consumption. The explanation for malaria-induced thrombocytopenia has been proposed by several authors (Coelho et al., 2013). There was some research suggested that the low level of platelet count in malaria might be because of apoptosis and/or activation of platelets. However, immune complexes elicited by malarial antigen may also be able to sequester injured platelets in the spleen and then be phagocytosed by splenic macrophages (Thapa et al., 2009; Lacerda et al., 2011; Zhan et al., 2019). The immune system attacks platelets resulting in immune thrombocytopenia (ITP). There are a few tests available to test antibodies against platelets (Michel et al., 2002). A majority of IgG subclasses are found bound to platelets in ITP. It may be useful to test for platelets-bound IgG in patients with thrombocytopenia.

*Plasmodium yoelii* and *P. chabaudai* are widely used as murine models to identify parasite induced immune responses. *Babesia rodhaini* and *B. microti* have been served as useful experimental model in mice for the analysis of human babesiosis (Rizk et al., 2020; Ji et al., 2021; Li et al., 2022). Anti-erythrocyte and anti-platelet autoantibodies producing is the crucial step between hematological complication and the host defense mechanisms after *Plasmodium* and *Babesia* spp. infection. To investigate the development of anti-erythrocyte and anti-platelet auto-antibodies and identify the related auto-antigens, in this study, the anti-erythrocyte and anti-platelet auto-antibodies were detected in *Plasmodium* and *Babesia* spp. infected mice, and the mechanism of antibody-mediated hemolytic anemia and thrombocytopenia were investigated.

## Materials and methods

### Mice ethics statement

From Central Institute for Experimental Animals (CIEA) in Japan, we purchased 6-week-old female ICR mice as well as C.B-17/Icr-scid/scid (SCID) mice. In accordance with the research protocol, the experimental animals were handled under the permit issued by Obihiro’s Animal Care and Use in Research Committee Promulgated by Obihiro University of Agriculture and Veterinary Medicine, Japan (Permit Number: 201109–5).

### Maintenance of the parasites and mice infections

*Plasmodium yoelii, P. chabaudi, B. rodhaini* and *B. microti* were maintained in mice by intraperitoneal (i.p.) passage as previously described (Li et al., 2012). SCID mice are severely combined immunodeficient mice. The weight of the thymus, spleen, and lymph nodes was less than 30% of normal, and histologically there were significant lymphocyte defects. To determine the role of B and T lymphocytes in the protection against infection with *P. yoelii, P. chabaudi, B. rodhaini* and *B. microti*, four groups of SCID mice were also infected with *P. yoelii, P. chabaudai, B. rodhaini* and *B. microti* by i.p. inoculation with 10^7^ of parasitized erythrocytes (pRBCs). At the same time, four groups of ICR mice were infected with *P. yoelii, P. chabaudai, B. rodhaini* and *B. microti* as mentioned above.

### Determination of survival rates and parasitemia

The survival rates and parasitemia of these groups of mice were monitored after *P. yoelii, P. chabaudi, B. rodhaini* and *B. microti* infection. The parasitemia of each mouse was tested by Giemsa-stained thin blood films. The blood samples from the ICR and SCID mice were serially collected every two days post-infection (dpi). The plasma was separated from RBCs by centrifuge (3000 r/min, 3 min), the RBCs and plasma were stored at -70°C until use.

### Detection of serum cytokines

The blood samples from the ICR and SCID mice infected with *P. yoelii, P. chabaudi, B. rodhaini* and *B. microti* were serially collected. The plasma was separated and used to detect the serum cytokines. A standard curve was used to determine the concentration of individual cytokines in the samples by using enzyme-linked immunosorbent assay (ELISA) kits. According to the manufacturer’s instructions, standard curves were prepared with mouse recombinant IFN-γ and interleukin (IL) -10 (Cusabio Biotech Co., Germany) according to the manufacturer’s instructions. Double-antibody one-step sandwich ELISA was used. The samples, standard products, and HRP labeled detection antibodies were added into the coated micropores pre-coated with antibodies, incubated and thoroughly washed. Substrate TMB was added to the micropores for color development. The depth of the color is positively correlated with the concentration of the substance in the sample. The absorbance (OD value) was measured at 450 nm wavelength and the sample concentration was calculated.

### Establishment of chronic infection

The mice should survive for more than 60 days to monitor the anti-erythrocyte and anti-platelet autoantibody level during infection. Therefore, we established the chronic infection and tried to extend the survival period of the *Plasmodium* and *Babesia* spp. infected mice. *P. yoelii, P. chabaudi, B. rodhaini *and *B. microti* were infected to 5 ICR mice for each group. The *Plasdodium* spp. infected-mice were treated by Artesunate, and the *Babesia* spp. infected-mice were treated by Diminazen for collecting the chronic stage serum. The parasitemia of each mouse was assessed every 2 days for 60 days. In order to determine the parasitemia percentage, thin blood smears were prepared, fixed in methanol, and stained for 30 minutes with 10% Giemsa solution. The level of parasitemia was estimated according to the matching method. A serial blood sample was collected from the ICR mice every two days. The plasma and RBCs were harvested and stored at -70°C until use.

### Platelets and red blood cells (RBCs) membrane isolation

Isolated platelets and RBC membranes were used for ELISA and two-dimensional LC and MS assays. To generate platelet-rich plasma (PRP), whole blood was centrifuged for 10 minutes at 200 x g. Platelets were acidified with citric acid 0.15 M until pH 6.4, then prostaglandin E-1 (PGE-1) was added at 1 mg/mL to prevent aggregation and activation.

After centrifugation, the pellet was resuspended in phosphate buffered saline (PBS). The whole blood was spun to separate packed red cells. To isolate host erythrocyte membrane, packed erythrocytes were extensively washed and lysed. The packed erythrocytes were washed in a ten-fold volume of 1X PBS. For the remaining steps, the washed cells were placed on ice. The washed RBC pellets were lysed with cold lysis buffer; after that, the lysis was spun on a Beckman Coulter ultracentrifuge, and five washes were performed after removing the supernatant. The resulting host membranes were collected and frozen at −70 °C.

### Detection of anti-erythrocyte and anti-platelet antibodies

The platelets and RBC membrane were washed two times, 100 ng of platelets and RBC membrane were coated in each well to detect of anti-erythrocyte and anti-platelet antibodies. After blocking, the mice serum collecting at all time points was added (50 µL/well, 100-time diluted) after incubating 1 hour and washing. We add 50 µL/well of a 1:2000 dilution in blocking buffer of HRP-labelled 2nd antibody. ELISA reader was used to read absorbance at 450 nm after incubation with stop solution. The quantity of erythrocyte and platelet-associated immunoglobulin IgM, IgG, IgG1, IgG2a, and IgG2b in plasma were determined by using the ELISA kit.

### Two-dimensional electrophoresis (2-DE) and Western blot analysis

The purified platelets and RBC membrane proteins were treated separately with DTT and iodoacetamide, and digested with trypsin. The sample was loaded on IPG strip, rehydration and focusing steps were run with isoelectric focusing carried out simultaneously over 17 hours at a total voltage of 35 kV/h. Second-dimension electrophoresis was performed at 200 volts for 45-50 minutes with Criterion precast gels. In the next step, colloidal Coomassie blue was used to stain the 2DE gels, followed by acetic acid to destain them. At the same time, 2DE gels were detected by Western blot using a mouse serum (diluted 1:100), which was generated in the laboratory by infecting with *Plasmodium* spp. and *Babesia* spp.

### Trypsin digestion and two-dimensional LC with MS/MS

In each gel, selected spots were manually removed and dehydrated with acetonitrile for 10 minutes before being dried with a Speed-Vac system. After overnight incubation, each dried protein spot was pipetted with trypsin solution and incubated. A Speed-Vac system was used to dry the supernatants after each step. The peptides were solubilized in 0.5% formic acid using an ultrahigh-performance liquid chromatography system. A reverse phase column Pepmap C18 was used for peptide separation. The most abundant peptides were analyzed by mass spectrometry (MS) using a continuous MS scan followed by 10 times analyses. The Mascot search engine was used to identify proteins in the NCBI Genbank database. The contaminants were excluded during the process.

### Statistical analysis

An analysis of one-way Analysis of variance test was used to determine if there were any significant differences between the means of all variables (GraphPad Prism 5; GraphPad Software, Inc.). The significance of P-values was denoted as follows: ns, non-significant; *, p ≤ 0.05; **, p ≤ 0.01; ***, p ≤ 0.001; and ****, p ≤ 0.0001.

## Results

### Thrombocytopenia and parasitemia after *Plasmodium* spp. and *Babesia* spp. infection

**Figure 1:**
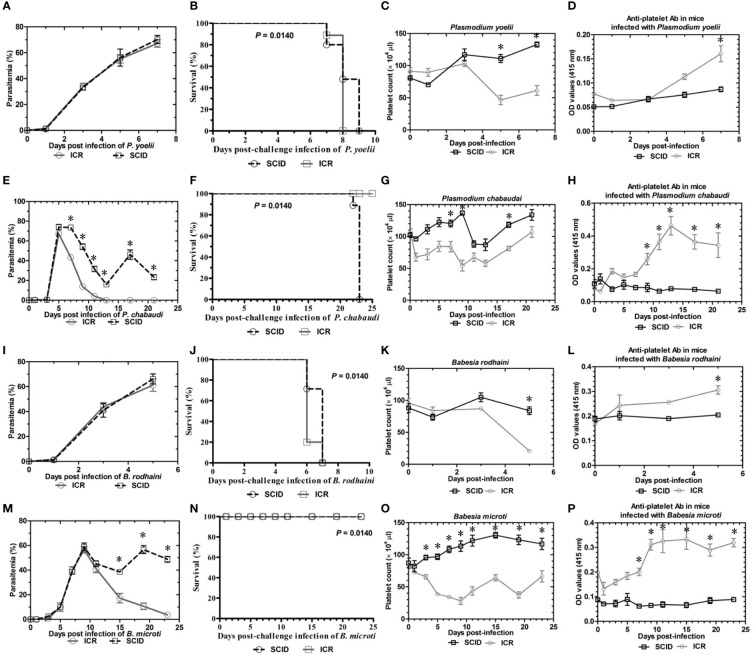
Parasitemia and survival rates, platelet counts and anti-platelet antibody of ICR mice and SCID mice after *Plasmodium yoelii, P. chabaudi, Babesia rodhaini* and *B. microti* challenge infection. **(A–D)** Parasitemia profiles of *P. yoelii, P. chabaudi, B. rodhaini* and *B. microti* infected ICR mice and SCID mice. **(B–H)** Survival rates of *P. yoelii, P. chabaudi, B. rodhaini* and *B. microti* infected ICR mice and SCID mice. **(I–L)** Platelet counts of *P. yoelii, P. chabaudi, B. rodhaini* and *B. microti* infected ICR mice and SCID mice. **(M–P)** anti-platelet antibody of *P. yoelii, P. chabaudi, B. rodhaini* and *B. microti* infected ICR mice and SCID mice. I.p. inoculations infected test mice with 10^7^ of parasitized erythrocytes (pRBCs). The results are expressed as mean value ± the SD for five mice. The significance of P-values was denoted as follows: ns, non-significant; *, p ≤ 0.05; **, p ≤ 0.01; ***, p ≤ 0.001; and ****, p ≤ 0.0001.

**Figure 2:**
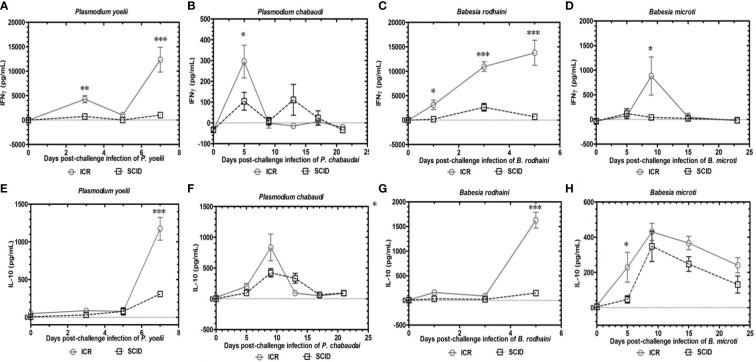
Kinetics of serum cytokines of ICR mice and SCID mice after *P. yoelii, P. chabaudi, B. rodhaini *and *B. microti* challenge infection. **(A–D)** Production of IFN-γ. **(E–H)** Production of IL-10 post challenge infection with *P. yoelii, P. chabaudi, B. rodhaini *and*B. microti*. The results are expressed as mean value ± the SD for five mice. The significance of P-values was denoted as follows: ns, non-significant; *, p ≤ 0.05; **, p ≤ 0.01; ***, p ≤ 0.001; and ****, p ≤ 0.0001.

To determine the role of B and T lymphocytes during lethal infection with *Plasmodium* spp. and *Babesia* spp, the immune sufficient ICR mice and SCID mice were infected with *P. yoelii*, *P. chabaudi*, *B. microti and B. rodhaini*. The percentage parasitemia, survival rate, platelet count and anti-platelet autoantibodies were tested (**Figures 1A–P**). In the group that infected with *P. yoelii* and *B. Rodhaini*, all the infected mice died within ten days (**Figures 1B, J**); parasitemia was similar in ICR and SCID mice (**Figures 1A, I**). The low survival rate was correlated with the high percentage of parasitemia. In the group that was infected with *P. chabaudi *and*B. microti*, all the mice were alive for more than 20 days (**Figures 1F, N**); parasitemia was similar in *P. chabaudi*-infected ICR and SCID mice (**Figures 1G, O**), the parasitemia in *B. microti*-infected ICR and SCID mice was significant different after 20 days infection (P<0.05). Both ICR and SCID mice developed rapid increases in parasitemia within one week of infection (**Figures 1E, M**). Compared with the SCID mice, high anti-platelet autoantibodies were found in *P. yoelii, P. chabaudi, B. microti* and *B. rodhaini* infected ICR mice (P<0.05). Furthermore, negative correlation existed between platelet count and anti-platelet in *P. yoelii* (**Figures 1C, D**)*, P. chabaudi* (**Figures 1G, H**)*, B. rodhaini* (**Figures 1K, L**) and *B. microti* (**Figures 1O, P**) infected ICR mice. The concentration of individual cytokines in Plasmodium and Babesia spp. infected mice has been tested (**Figures 2A–G**). Likewise, detectable IFN-γ and IL-10 levels were lower in SCID mice (P<0.01) than those detected in ICR mice at days 5 and 7 post challenge with *P. yoelii and B. rodhaini* (**Figure 2H**). According to these findings, the anti-platelet autoantibodies induced by *Plasmodium* spp. and *Babesia* spp infection might be impaired by B and T lymphocytes.

### The *Plasmodium* spp. and *Babesia* spp. infection induces the production of anti-erythrocyte and anti-platelet autoantibodies

**Figure 3:**
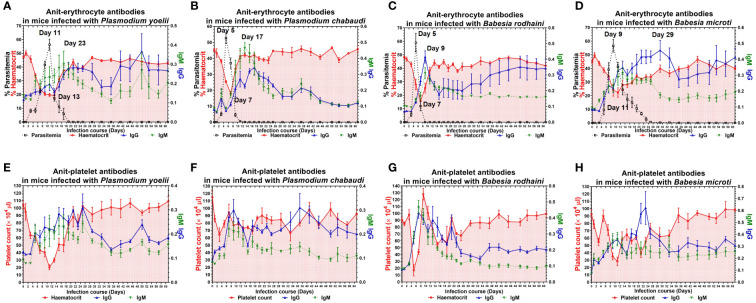
The anti-erythocyte and anti-platelet antibodies of chronically *P. yoelii, P. chabaudi, B. rodhaini* and *B. microti*. infected ICR mice. **(A–D)** The parasitemia, hematocrit, and the production of anti-erythocyte IgG and IgM in ICR mice challenge infection of *P. yoelii, P. chabaudi, B. rodhaini *and *B. microti*. **(E–H)** The platelet counts and the production of anti-platelet IgG and IgM in ICR mice challenge infection of *P. yoelii, P. chabaudi, B. rodhaini* and *B. microti*. Test mice with chronic *P. yoelii, P. chabaudi, B. rodhaini *and*B. microti* infection, the *Plasmodium* spp. infected mice were treated with artesunate, and the *Babesia* spp. infected mice were treated with diminaze for collecting the chronic stage sera. The parasitemia of each mouse was examined every 2 days for 60 days. The results are expressed as mean value ± the SD for five mice.

To examine the possible contribution of anti-erythrocyte and anti-platelet autoantibodies in thrombocytopenia and hemolytic anemia caused by lethal infection with *Babesia* spp. and *Plasmodium* spp. The chronic *Babesia* spp. and *Plasmodium* spp. infections were established by administering artesunate and diminaze to infected-ICR mice. The trend of parasitemia was similar in *Babesia* spp. and *Plasmodium* spp. infected mice, and hematocrit and parasitemia were negatively correlated (**Figures 3A–H**). The IgM and IgG reached to the highest level after reaching high parasitemia (**Figures 3A–D**). These results indicated that *Plasmodium* spp. and *Babesia* spp. infection lead to the destruction of RBCs and the production of anti-erythrocyte autoantibodies may not be the important reason for anemia. The platelet counts significantly decreased in the mice infected with *Plasmodium* spp. and *Babesia* spp. Moreover, IgM and IgG levels negatively correlated with platelet count (**Figures 3E, F**). Thus, anti-platelet auto-antibodies may be the cause of thrombocytopenia.

### The IgG isotypes of anti-erythrocyte and anti-platelet autoantibodies

**Figure 4:**
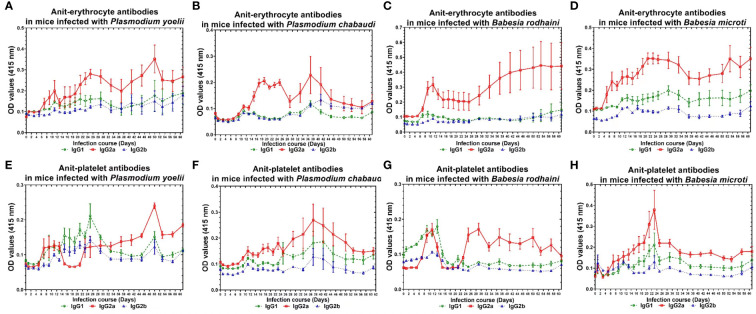
The production of anti-erythocyte and anti-platelet IgG1, IgG2a, IgG2b of of chronically *P. yoelii, P. chabaudi, B. rodhaini* and *B. microti* infected ICR mice. **(A–D)** The production of anti-erythocyte IgG1, IgG2a, IgG2b in ICR mice challenge infection of *P. yoelii, P. chabauai, B. rodhaini *and* B. microti* **(E–H)**. The anti-platelet IgG1, IgG2a, IgG2b in ICR mice challenge infection of *P. yoelii, P. chabaudi, B. rodhaini* and *B. microti*. Test mice with chronic *P. yoelii, P. chabaudi, B. rodhaini* and *B. microti*. infection, the *Plasmodium* spp. infected mice were treated with artesunate, and the *Babesia rodhaini* infected mice were treated with diminaze for collecting the chronic stage sera. The parasitemia of each mouse was examined every 2 days for 60 days. The results are expressed as mean value ± the SD for five mice.

The infected mice produced a high amount of anti-platelet and anti-erythrocyte IgG2a and a low amount of IgG1 and IgG2b after *Plasmodium* spp. and *Babesia* spp. infection. The anti-erythrocyte IgG2a was detectable ten days after infection and peaked between 22 and 30 days and 40 and 50 days (**Figures 4A–D**) in *plasmodium* spp. and *Babesia* spp. infection. The anti-platelet IgG2a was detectable within six days after *Plasmodium* spp. and *Babesia* spp. infection. The anti-platelet IgG2a reached to high level at 24 days and 48 days after illness in *P. yoelii*-infected mice. The *P. chabaudi-*infected mice had elevated levels of anti-platelet antibody at 40 days post-infection. Furthermore, anti-platelet antibody also reached the peak at 22 days post-infection in *B. microti* infected mice. However, the *B. rodhaini*-infected mice developed low moderate levels anti-platelet IgG2a (**Figures 4E–H**).

### Identification of murine erythrocyte and platelet auto-antigens

**Figure 5:**
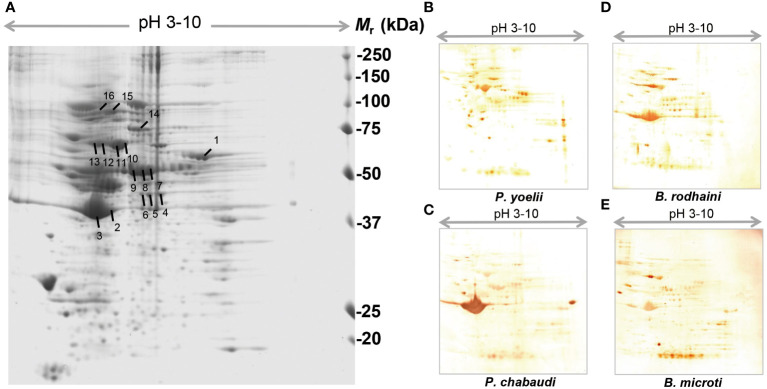
Reference image 2DE map of differentially expressed platelet proteins with marked selected spots and Western blot analysis of murine platelet proteins probed with auto-antibodies induced by *P. yoelii, P. chabaudi, B. rodhaini* and *B. microti*. infection. **(A)** Representative 2DE map of murine platelet proteins. **(B)** Western blot analysis of murine platelet proteins probed with auto-antibodies induced by *P. yoelii* infection. **(C)** Western blot analysis of murine platelet proteins probed with auto-antibodies induced by *P. chabaudi* infection. **(D)** Western blot analysis of murine platelet proteins probed with auto-antibodies induced by *B. rodhaini* infection. **(E)** Western blot analysis of murine platelet proteins probed with auto-antibodies induced by *B. microti* infection. A representative 2DE map of murine platelet proteins was obtained by performing the first dimension (IEF) on IPG strips pH 3–10 and the second dimension on 4–12% gradient SDS-PAGE gels. The protein spots were visualized by Coomassie blue staining. The indicated spots were excised from the gel and identified by MS/MS.

**Figure 6:**
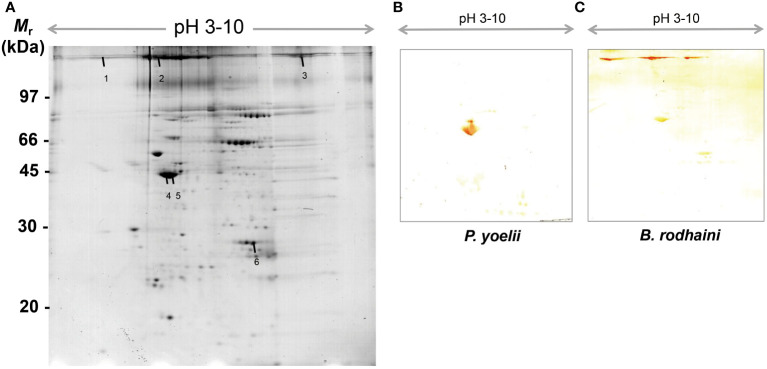
Reference image 2DE map of differentially expressed erythrocyte proteins with marked selected spots and Western blot analysis of murine erythrocyte proteins probed with auto-antibodies induced by *P. yoelii* and *B. rodhaini* infection. **(A)** Representative 2DE map of murine erythrocyte proteins. **(B)** Western blot analysis of murine erythrocyte proteins probed with auto-antibodies induced by *P. yoelii* infection. **(C)** Western blot analysis of murine erythrocyte proteins probed with auto-antibodies induced by *B. rodhaini* infection. Representative 2DE map of murine platelet proteins obtained by performing the first dimension (IEF) on IPG strips pH 3–10 and the second dimension on 4–12% gradient SDS-PAGE gels. The protein spots were visualized by Coomassie blue staining. The indicated spots were excised from gel and identified by MS/MS.

In order to further investigate and understand the mechanism of autoimmune antibody-mediated thrombocytopenia and hemolytic anemia, we isolated the platelets and RBC membrane proteins followed by identification of the autoimmune antibody-binding proteins by 2DE and Western blot analysis. 2DE analysis of platelets samples showed differentially expressed spots (**Figure 5A**). In **Figures 5B–E**, representative gel images for each group are shown. A total of sixteen spots were selected for mass spectrometry analysis. 2DE image analysis of RBC membrane showed differentially expressed spots (**Figure 6A**). Each experimental group is represented by a gel image in **Figures 6B, C**. For mass spectrometry, six spots were chosen. Overall, the results showed that many host polypeptides are bound to autoimmune antibodies specifically (P<0.05).

### Comparative analysis of the antibody-binding proteins

The selected spots were analyzed by 2D LC-MS/MS after trypsinization to identify the autoimmune antibody-binding proteins. Our study combined Western blot, 2-DE, and proteomic analysis. All of the above proteins have been identified *via* analysis of the antibody-binding proteins. As shown in **Tables 1** and **2**, the MS/MS spectra have been analyzed. Masses of the peptides identified by LC-MS/MS were compared with sequences from the National Center for Biotechnology Information database (NCBI: http://www.ncbi.nlm.nih.gov/), separately. By using the ion score, we compare the fragment ions to all tryptic peptides calculated from parasites and mice. Actin of murine erythrocyte and platelet is a common auto-antigen in *Plasmodium* spp. and *Babesia* spp. infected mice.

**Table 1:** Protein identification of murine erythrocyte proteins probed with anti-erythrocyte auto-antibodies.

Spot no.	Accession no.	Protein name	Theoretical *M*_r_ (Dr)/*pI*	Sequence coverage (%)	MASCOT value
1	gi|187956529	Spectrin alpha 1	280931/4.94	20%	156
2	gi|187956529	Spectrin alpha 1	280931/4.94	17%	149
3	gi|187956529	Spectrin alpha 1	280931/4.94	18%	141
4	gi|469566230	beta actin	40847/5.56	53%	145
5	gi|512956198	actin	41995/5.29	49%	130
6	gi|156257635	beta-globin	15838/7.86	62%	91

**Table 2:** Protein identification of murine platelet proteins probed with anti-platelet auto-antibodies.

Spot no.	Accession no.	Protein name	Theoretical *M*_r_ (Dr)/*pI*	Sequence coverage (%)	MASCOT value
1	gi|148683477	fibrinogen	63570/6.95	26	87
2	gi|49868	beta-actin	39446/5.78	39	104
3	gi|74213524	actin	42066/5.30	73	207
4	gi|568930542	alpha-enolase	47640/6.30	40	139
5	gi|568930542	alpha-enolase	47640/6.30	36	123
6	gi|568930542	alpha-enolase	47640/6.30	46	120
7	gi|33859809	fibrinogen beta chain	55402/6.68	26	100
8	gi|33859809	fibrinogen beta chain	55402/6.68	34	168
9	gi|33859809	fibrinogen beta chain	55402/6.68	25	97
10	gi|26341396	serum albumin precursor	67013/5.49	21	108
11	gi|26341396	serum albumin precursor	67013/5.49	29	166
12	gi|74142813	heat shock cognate 71 kDa protein	50547/6.17	27	87
13	gi|178847300	70kDa heat shock cognate protein	59895/5.91	32	116
14	gi|1430883	zyxin	62063/6.47	20	74
15	gi|148670554	valosin containing protein	91675/5.26	27	127
16	gi|149045716	valosin-containing protein	76799/5.49	43	229

## Discussion

Malaria and babesiosis continue to be important diseases in the world. The varied presentations of these diseases and their diversity in terms of hematological manifestations have been well endowed in literature (Zhan et al., 2019; Wang et al., 2020; Varshavsky et al., 2021; Voisin et al., 2021). The most common hematological complications of malaria and babesiosis are thrombocytopenia and anemia. Anemia is caused by a various of pathophysiologic mechanisms, including accelerated RBCs removal by the spleen, obligatory RBCs destruction at parasite schizogony, and ineffective erythropoiesis (Nie et al., 2021; Rizk et al., 2021; Rizk et al., 2022). Recent advancements have shown that a variety of cytokine dysregulations are indeed vital participants in inducing and accelerating the pathogenesis of hemolysis in malaria and babesiosis. They include a significant increase in IFN-γ, IL-6 and IL-1 and a decrease in IL-10 and IL-12 levels. In patients with malaria and babesiosis, autoimmune hemolytic anemia (AIHA) has been described previously (Narurkar et al., 2017; Santos et al., 2020; Rajapakse and Bakirhan, 2021). Several parasite and virus infections have been reported to be associated with AIHA, such as influenza virus, *Leishmania* species, hepatitis virus, and cytomegalovirus. Multiple studies have documented the high frequency of thrombocytopenia in malaria patients (Rodríguez-Morales et al., 2005; Coelho et al., 2013). For more than four decades, researchers have investigated the pathogenesis of malaria thrombocytopenia, but it remains unclear how it occurs (McMorran et al., 2009). According to some studies, malaria may cause low platelet counts due to activation or apoptosis of platelets, which prevents the immune system from removing them. Nevertheless, malarial antigens have also been implicated in sequestering injured platelets in the spleen due to immune complexes formed. In addition, there are some evidences of platelet-associated IgG involvement in malaria thrombocytopenia. Immune-mediated hemolytic anemia and thrombocytopenia in malaria and babesiosis has gathered more attentions in recent years (Lacerda et al., 2011).

We investigated the autoimmune-mediated hemolytic anemia and thrombocytopenia during *Plasmodium* spp. and *Babesia* spp. infection in this study. High levels of anti-platelet auto-antibodies were found in *P. yoelii, P. chabaudi, B. rodhaini and B. microti* infected ICR mice. In contrast, SCID mice displayed lower level of anti-platelet auto-antibodies. There was obvious relation between platelet count and anti-platelet. According to the findings of a previous study, acute malaria and babesiosis infection are associated with AIHA. B and T lymphocytes are the important inducers of the immune effector mechanisms, which are needed for initial control of *Plasmodium* spp. *and Babesia* spp. infection. Therefore, the SCID mice are unable to produce anti-platelet auto-antibodies (Watier et al., 1992; Wheeler et al., 2011; Aschermann et al., 2013). Our data indicate that the absence of B and T lymphocytes impaired the production of anti-platelet auto-antibodies. There are a few possible reasons of the presence of anti-platelet auto-antibodies. Such as the erythrocyte share some similar peptides with platelet, which induced auto-antibodies against both, or the broken erythrocyte induced disrupt of some platelet, which consequently induced anti-platelet auto-antibodies thus lead to more platelet disruption and more antibody. We found that ICR mice infected with *Plasmodium* and *Babesia* species emitted significantly higher levels of IL-10 and IFN-γ. It is a strong support for the idea that the timing and magnitude of specific cytokines influence the severity of malaria and babesiosis. It is necessary to study the cytokine production in *Plasmodium* spp. and *Babesia* spp. infected mice.

In addition, chronic infection with *Plasmodium* spp. and *Babesia* spp. was established after the *Plasdodium* spp. infectedmice were treated by artesunate, and the *Babesia* spp. infectedmice were treated by diminaze. The trend of parasitemia was similar in *Plasmodium* spp. and *Babesia* spp. infected mice, a negative correlation was observed between parasitemia and hematocrit. Moreover, The IgM and IgG reached to the highest level after the day of high parasitemia (Boes et al., 2000). These observations suggest that the production of anti-erythrocyte autoantibody is not the main reason of anemia. Generally, platelet antibodies in ITP are IgG or IgM, but IgA and IgE have also been reported (Klaassen et al., 1989). IgG are responsible for interacting with macrophages in the reticuloendothelial system when antibodies bind to platelets. Complement-mediated lysis can also remove antibodies-sensitized platelets from circulation. Therefore, the platelet-associated IgG and IgM were comparably elevated in the majority of *Plasmodium* spp. and *Babesia* spp. infected mice, and the IgG might be the majority of platelet antibodies in ITP. In this study, the anti-platelet IgG2a and low amounts of IgG1 and IgG2b also was detected after *Plasmodium* spp. and *Babesia* spp. infection.

The autoimmune antibody-binding proteins were identified in the study. The autoimmune antibody-binding proteins identified for platelet and RBC membrane were similar in both subcellular location and function categories. In contrast, membrane-associated cytoskeleton proteins from platelet and RBCs membrane was found (**Tables 1**, **2**). Though we could not rule out that the actual number of platelet and RBC membrane proteins, which were more than that of the membrane-associated cytoskeleton proteins may affect the production of autoimmune antibodies. The membrane associated cytoskeleton proteins triggering the autoimmune response in *Plasmodium* spp. and *Babesia* spp.infected mice require further characterization.

In conclusion, we have demonstrated that the autoimmune response is elicited during *Plasmodium* spp. and *Babesia* spp. infection. The autoimmune antibody may participate in thrombocytopenia and hemolytic anemia and regulate the autoimmune response. As a result of this research, we can develop an effective babesiosis and malaria therapeutic that modulates autoimmune responses for overcoming infection. Understanding of the effector molecules that inhibit autoimmune responses may provide important clues for future infection control strategies. In addition to antibiotics for the treatment of malaria and babesiosis, ITP treatment should be initiated in severe cases.

## Data availability statement

The datasets presented in this study can be found in online repositories. The names of the repository/repositories and accession number(s) can be found in the article/supplementary material.

## Ethics statement

The animal study was reviewed and approved by Animal Care and Use in Research Committee Promulgated by Obihiro University of Agriculture and Veterinary Medicine, Japan (Permit Number: 201109–5).

## Author contributions

XX, SC, and MZ designed the study. MZ, SC carried out the experiments. OK and YN provide technical support. MZ, SC, JX, SJ, SZ, and XX wrote and read the manuscript, and all authors reviewed the manuscript. All authors contributed to the article and approved the submitted version.
